# Probabilistic Prognosis of Environmental Radioactivity Concentrations due to Radioisotopes Discharged to Water Bodies from Nuclear Power Plants

**DOI:** 10.3390/toxics5040032

**Published:** 2017-11-15

**Authors:** Juan Tomás Zerquera, Juan C. Mora, Beatriz Robles

**Affiliations:** 1 Center for Radiation Protection and Hygiene (CPHR), Calle 20 No. 4113 entre 41 y 47, Miramar, Playa, Havana 12500, Cuba; jtomas@cphr.edu.cu; 2 Radiological Protection of the Public and the Environment Unit, CIEMAT, Avda, Complutense, 40, 28040 Madrid, Spain; 3 Nuclear Safety Council (CSN), Calle Pedro Justo Dorado Dellmans, 11, 28040 Madrid, Spain; beatriz.robles@csn.es

**Keywords:** environmental monitoring program, nuclear power plants, radioecological modelling, normal discharges

## Abstract

Due to their very low values, the complexity of comparing the contribution of nuclear power plants (NPPs) to environmental radioactivity with modeled values is recognized. In order to compare probabilistic prognosis of radioactivity concentrations with environmental measurement values, an exercise was performed using public data of radioactive routine discharges from three representative Spanish nuclear power plants. Specifically, data on liquid discharges from three Spanish NPPs: Almaraz, Vandellós II, and Ascó to three different aquatic bodies (river, lake, and coast) were used. Results modelled using generic conservative models together with Monte Carlo techniques used for uncertainties propagation were compared with values of radioactivity concentrations in the environment measured in the surroundings of these NPPs. Probability distribution functions were inferred for the source term, used as an input to the model to estimate the radioactivity concentrations in the environment due to discharges to the water bodies. Radioactivity concentrations measured in bottom sediments were used in the exercise due to their accumulation properties. Of all the radioisotopes measured in the environmental monitoring programs around the NPPs, only Cs-137, Sr-90, and Co-60 had positive values greater than their respective detection limits. Of those, Sr-90 and Cs-137 are easily measured in the environment, but significant contribution from the radioactive fall-out due to nuclear explosions in the atmosphere exists, and therefore their values cannot be attributed to the NPPs. On the contrary, Co-60 is especially useful as an indicator of the radioactive discharges from NPPs because its presence in the environment can solely be attributed to the impact of the closer nuclear facilities. All the modelled values for Co-60 showed a reasonable correspondence with measured environmental data in all cases, being conservative in two of them. The more conservative predictions obtained with the models were the activity concentrations in the sediments of a lake (Almaraz) where, on average, values two times higher were obtained. For the case of rivers (Ascó), calculated results were adequately conservative—up to 3.4 times on average. However, the results for coasts (Vandellos II) were in the same range as the environmental measurements, obtaining predictions that are only—at maximum—1.1 times higher than measured values. Only for this specific case of coasts could it be established that the models are not conservative enough, although the results, on average, are relatively close to the real values.

## 1. Introduction

In carrying out prospective assessments of the radiological impact of routine releases from a variety of industrial facilities, including nuclear power plants (NPPs), the European Union (EU), the International Atomic Energy Agency (IAEA), and others have developed models which are widely accepted.

In particular, the IAEA published the Safety Report Series No. 19 (SRS-19) document [1], where a graded approach to be used for assessing the radiological impact of discharges to the environment is described. In this approach, the use of intentionally conservative simple models is recommended for the first stages of the assessment. These models use a very limited quantity of information on the characteristics of the releases or the affected environment. More complex models are also described in this document which can be used if needed. Additionally, SRS-19 provides many values to be used by default in some parameters of those models, which are assumed to be conservative in any situation. Here conservative means that there is a low risk of underestimating the concentration values which can be found in real measurements in the environment after routine discharges, within the degree of variability.

Models or parameters can be refined with the intention of achieving a better correspondence of modelled values with measurements. However, this refinement should be ideally complemented with an evaluation or a discussion of the uncertainty associated with the assessment. This uncertainty involves many components [2,3], one of them being the uncertainty associated with the parameters used in the mathematical models. This component of the uncertainty is usually represented by the shape and size of a probability distribution function (pdf), then being evaluated as an intrinsic part of the estimations of effective dose to the representative person [4].

Measurements of the environmental concentrations of radionuclides are usually provided as a central value (typically a mean value) and its associated uncertainty, which includes the standard deviation and other uncertainties associated with the method of measurement, such as the uncertainty of the standard used for the calibration, or the variability of the measured magnitude, among others. Therefore, the values obtained in environmental monitoring programs around nuclear installations are usually described as ranges instead of single values, giving a probability (usually a 95% for a coverage factor k = 2) of finding the real value within the range.

Those parametric uncertainties were also included in the results of generic simple models, in order to analyse how it affects a comparison with the ranges of measured values of radionuclide concentrations in a specific compartment of the environment. For this, IAEA’s SRS-19 models were used together with public data on discharges from some Spanish NPPs to obtain prospective modelled values. Finally, a comparison of those modelled results with the measured ranges obtained in the environmental monitoring programs performed in the surroundings of these NPPs was carried out. Specifically, an environmental compartment affected by a cumulative process—sedimentation—was selected, due to the very low level of discharges produced from these installations. Given the conservatism of the models used, it is expected that the modelled results will be significantly above measured concentrations.

The present paper shows the results of those comparisons.

## 2. Materials and Methods

Many possible scenarios could be taken into account for assessing the impact of Spanish NPPs on the environment. However, it was assumed that the main differences would appear in the water bodies where each NPP releases their liquid effluents. Three different scenarios based on real NPPs were considered:

1. Release to a reservoir. The case of Almaraz NPP was taken as representative;
2. Release to coastal waters. Vandellós II NPP was taken this time as example; and
3. Release to a river. Ascó NPP was used as representative of this situation.

The data for the quantities of each radionuclide annually discharged to the atmosphere and to water bodies (the source term) from Spanish NPPs (see Table 1) was taken from the published European Union Radiation Protection Series Report No. 143 [5].The dispersion models described in SRS 19 were calculated using the Código de cRiba para la evaluaciÓn de iMpacto (CROM) code [6,7].

CROM was designed to automate the calculation of radionuclide concentrations in different compartments of the environment and their transfer to the human food chain, as well as to estimate the effective dose for humans, using generic models for transport, dilution, and transfer from SRS 19. In order to estimate the radionuclide concentrations, the quantities and types of discharged radionuclides (the source term), the mode and characteristics of the discharge, and the receptor points need to be specified. The atmospheric dispersion model is a Gaussian plume model, accounting for the effects of buildings in the vicinity of the release and the effect of the roughness of the ground, designed to assess annual averaged radionuclide concentrations in the air. The surface water models account for dispersion in rivers, lakes, estuaries, and sea coasts. These aquatic models are based on analytical solutions of advection–diffusion equations describing radionuclide transport in surface water with steady-state uniform flow conditions. All the models contain many default values that can be used in the absence of local specific information. The terrestrial food chain models accept inputs of radionuclides from both the atmosphere and the hydrosphere. The process of radioactive decay and build-up is taken into account. The estimated radionuclide concentrations in air, soil, sediment, food, and water (calculated for 30-years of discharge) are combined with the annual rates of intake, the occupancy factors, and the appropriate dose conversion coefficients to obtain the maximum human effective dose for the representative person. Version 8 of CROM [8] allows the propagation of parameters’ uncertainties in the models by using Monte Carlo methods. This last version, CROM 8, implements a default database with data for 162 radionuclides.

The comparison of prospective modelled results of air concentrations obtained from atmospheric discharges with measured data was assumed to be not possible for the given conditions of an NPP, as the discharges to the atmosphere from nuclear power plants are usually very low, suffering an additional dilution in the atmosphere, which results in concentrations in the air lower than the usual detection levels for most of the radionuclides. The same can be said for the concentrations in water due to routine discharges from NPPs. However, concentration mechanisms allow the measurement of some radionuclides by using nuclear measurement techniques with typical detection levels in those media, as for example accumulation in bio-indicators, on soils, or in bottom sediments. For this reason, the focus in this work was put on the prospective assessments and comparison with measured radioactivity concentrations in one of them: bottom sediments, as a result of environmental cumulative processes in liquid bodies. Moreover, in this particular case, published measured concentration values were mostly above the detection limits for those three radionuclides.

For comparing the results of the prospective assessments with the values measured in the environment, data of measurements of radionuclide concentrations in sediments collected in the vicinity of selected Spanish NPPs were used, covering three different aquatic environments: reservoirs (or lakes), rivers, and coastal waters. Measurements in the environment, obtained from routine environmental radiological monitoring programs established around all of the Spanish NPPs, are published annually by the Spanish Nuclear Safety Council (CSN), including values for radionuclide concentrations in bottom sediments of those water bodies where liquid effluents are released. For the tests carried out in this study, the period 1999–2007 was used [9,10,11,12,13] (Table 1).

For prospective assessments, data on radioactive discharges (the source term) were defined as a stochastic variable for the input data. Information on releases was derived assuming triangular distributions for the input data of release rates. This assumption was made because the information used for the discharges from each NPP consists of averaged values of the discharges for each year during a period of five consecutive years. Triangular distribution is typically used as a subjective description of a population for which there is only a limited set of sample data where a range and best estimate of the value can be identified. For triangular distributions, the minimum and maximum values of releases in the period studied were taken as parameters, using the mean value as the central tendency estimator. Table 1 shows source term data assumed for each studied NPP.

For modelling the concentrations in bottom sediments, models and distribution coefficients (Kd) taken from SRS 19 [1] were used. In particular, for the case of freshwater scenarios (Almaraz—dammed reservoir and Ascó—river), Kd values were introduced as stochastic input parameters lognormally distributed, according to data provided by the reference. For the remaining parameters used for calculations of radionuclide concentrations in bottom sediments, default values provided in the SRS 19 were adopted.

The use of conservative assumptions in the models assures that prospective assessments of concentrations in the different environmental objects should be above measured values in any real situation.

## 3. Results and Discussion

### 3.1. Reservoirs (Almaraz NPP)

Table 2 collects the modelled results obtained in this case, given the above explained assumptions, together with the range of measured data in the bottom sediments of the reservoir receiving the discharges from this specific NPP. For the case of a reservoir or a lake, a single box model which considers instantaneous and homogeneous mix is used. Therefore, no dependence on the point of measurement is needed. Additionally, equilibrium state is conservatively assumed. In this particular case, measured Sr-90 values in the environment were always below the detection limits, and therefore a comparison was not possible. Although the results of the prospective assessments were expected to be well above the measured values due to the conservatism of the models, it was not the case in all the comparisons.

For the case of Cs-137, both calculated and measured values are very close. This effect can be explained by the contribution of radioactive precipitations (global fallout) to the activity concentration of Cs-137 on the soils, and the subsequent cumulative processes due to the wash out and the erosion of the surface soils, which contaminates the water of the rivers, and finally the continuous sorption and deposit of sediments into the bottom. The same results were observed by several authors who provided a similar answer to this observation [14,15].

**Figure 1:**
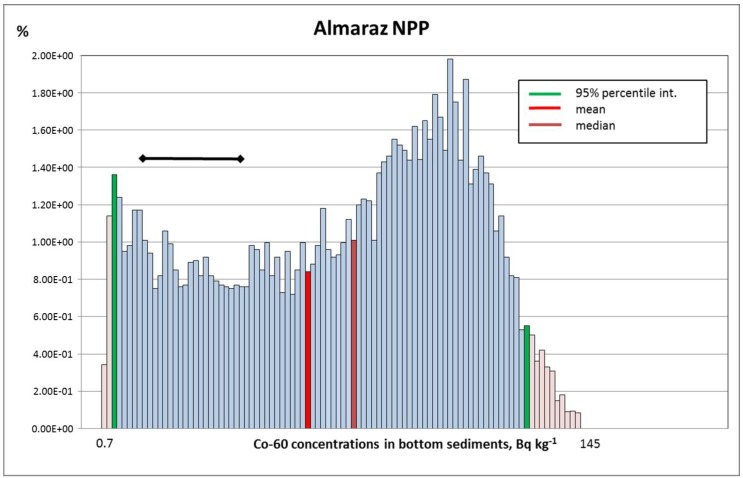
Co-60 activity concentrations calculated in bottom sediments (Bq kg^−1^) at Arrocampo reservoir (Almaraz NPP). The range of the published measurements is represented with the black segment.

On the other hand, Co-60 is a relatively short-lived radionuclide (T1/2 = 5.27 y), and therefore any past contributions have almost disappeared and any existence of this radionuclide in the environment can be solely attributed to the contribution of the NPP into that environmental compartment. Table 2 and Figure 1 show the results obtained for this particular radionuclide, where an acceptable correspondence is observed, the modelled values (probability distribution shown in the figure) being adequately conservative compared with the measured values (black segment), with differences of around almost one order of magnitude, as expected.

### 3.2. Coastal Waters (Vandellós II NPP)

In this case, Sr-90 was also included in the assessments of liquid releases, as the radionuclide is reported in the measurements of activity concentrations in bottom sediments above the detection limit.

The model applied for calculations of concentrations in coastal waters uses three parameters dependent on the location of both the release and the receptor points [1]. For the scenario covering this case (Vandellós II NPP), y0 = 0 was assumed (i.e., the release is produced on the shoreline, where the measurements are also performed at a certain distance x). Information on the location of sediment sampling points for monitoring was not provided in the public reports. Therefore, prospective calculations were carried out in four different points covering the applicability range of the model. Points were identified as Sea 1 to Sea 4 (see Table 3), the results in Sea 2 being the more conservative for all the radionuclides.

For Cs-137 and Sr-90, calculations resulted in values below measured values. However, the same issue discussed in the Almaraz NPP regarding the contribution of the global fall-out was observed in this site. For instance, estimations performed in the 1960s of the Sr-90 fall-out at 40° N (Vandellós’ latitude) were nearly 400 Bq m^−2^ [16], which, corrected for 47 years of radioactive decay (T1/2(Sr-90) = 28.8 y), would return a value of around 130 Bq m^−2^. Using typical values for soil density (1300 kg/m^3^) and a 5 cm depth (usually used for considering the leaching of a deposition in the surface of a soil), a value or 1.99 Bq kg^−1^ is obtained. This value is in very good agreement with the measured values for this particular radionuclide (1–3 Bq kg^−1^), supporting the hypothesis.

**Figure 2:**
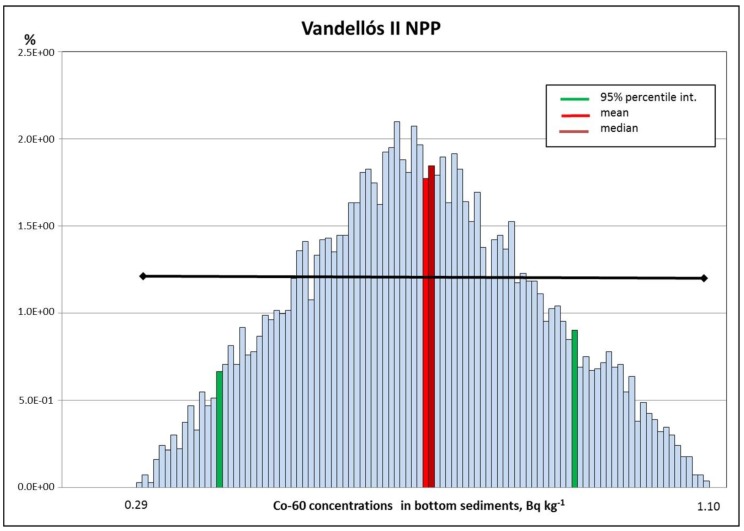
Co-60 concentrations calculated in bottom sediments (Bq kg^−1^) at the coastal line (Vandellos II). Black segment shows the range of the published measurements.

Although there are huge uncertainties associated with the lack of precise information on location of sampling points, a reasonable correspondence is observed between calculated results and measured data for the case of Co-60 (see Table 3 and Figure 2). Again, the presence of this radioisotope in bottom sediments can be only associated with releases from the facility. There is observed a lack of the conservatism expected from the prospective model in any case.

### 3.3. Rivers (Ascó NPP)

The model used for rivers [1] uses the distance from the release point to the shore location downstream and the consideration of the measurement point being located at the same shore or at the opposite shore where discharge is produced. In this case, the information for the exact location of bottom sediments’ sampling points was not provided. Five points at representative distances downstream (River 1 to River 5) were considered. Table 4 shows the distances used for those points together with the main results of the calculations and the range of the measured data.

As complete lateral mixing distance for the studied case is 14,000 m, significant differences are observed in the modelled values for points located on the opposite shore or on the same shore where discharge is produced (see Table 4). As the exact location of sampling points is unknown, the most conservative assumption is to consider that they are located on the same shore where the discharge is produced. Therefore, the modelled values obtained using this assumption were finally compared with the measurement results. Additionally, in this particular case, for those radionuclides affected by global fallout (i.e., Cs-137 and Sr-90), modelled values were below measured values in all cases. Other studies [17] performed in the same river also concluded that levels of Sr-90 and Cs-137 in water were unaffected by the presence of Ascó NPP, attributing this radioactivity to the fall-out of former nuclear weapons tests.

**Figure 3:**
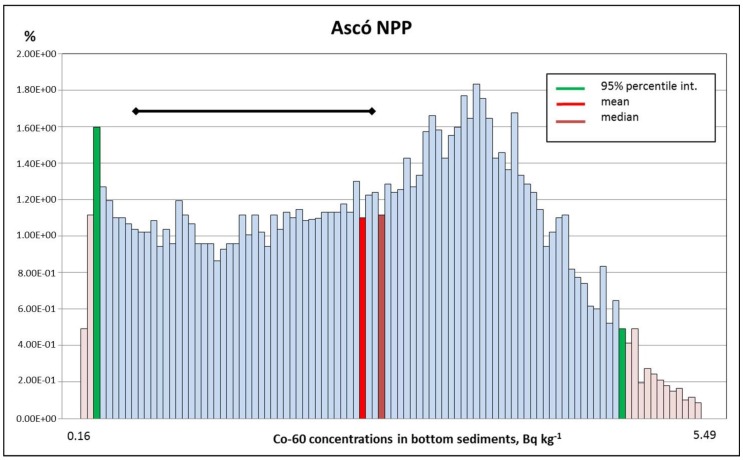
Co-60 concentrations calculated in one point in bottom sediments (Bq kg^−1^) at the Ebro river (Ascó). Black segment represents the range of the published measurements.

For the case of Co-60, the same arguments provided above are applicable. As can be seen in Table 4 and Figure 3, a reasonable correspondence between calculated and measured values was evidenced, with a reasonable degree of conservatism.

## 4. Conclusions

The comparison of prospective calculations of environmental radioactivity concentrations—which would be caused by radioactive discharges from NPPs—with measured values is obviously interesting for ensuring an adequate degree of conservatism of the models commonly used for the assessment and protection against radiation of the persons and the environment around nuclear power plants. However, due to the very low values of discharged radioactivity, the comparison of the contribution of NPPs to the environmental radioactivity with modeled values is generally complex. In this study, IAEA-recommended models for the assessment of routine discharges—assumed to be appropriately conservative in all cases—were used.

Only measurements in some environmental compartments where cumulative processes occur were above detection limits in all the studied cases, and could therefore be used for comparisons. In particular, comparisons of prospectively modelled values with measured concentrations were only performed for bottom sediments. Studies were carried out in three Spanish NPPs considered to be representative of the different aquatic bodies where discharges can be produced: Almaraz, Vandellós II, and Ascó. Specifically, three radionuclides were considered in all the cases for both the source term and for the values measured in the environment: Cs-137, Sr-90, and Co-60.

For prospective assessments of radioactive concentrations in bottom sediments, data on the source term (quantities discharged from the NPP to the aquatic receptor body) published by the European Union, together with dispersion models accepted by the IAEA for this specific situation were used. CROM code which implements those mathematical models and Monte Carlo methods for uncertainties propagation were used for the stochastic calculations. Conservative assumptions were also used for selecting the locations where values were modeled.

The results from radiological environmental monitoring programs around the Spanish NPPs—publicly distributed by the Spanish nuclear regulatory body (CSN)—were used for obtaining the measured radioactivity concentrations in the bottom sediments.

In the comparisons, modelled results for both Cs-137 and Sr-90 remained below or very close to measured values in all three cases. In fact, for one of them (Almaraz), values for Sr-90 measurements were below the detection limits. This phenomenon has been attributed in this study—and also in other studies—to the ubiquitous presence of these radionuclides in the environment, caused mainly by the global fall-out. A good agreement was obtained between the estimation of such fall-out from nuclear weapons tests at the location of the NPPs and reported measurements. In conclusion, the values obtained in the measurements of radioactivity concentrations in the environment around the NPPs cannot be simply attributed to the continuous discharges produced in normal operation. In other words, routine releases from nuclear facilities induce increases in environmental concentrations of Cs-137 and Sr-90 which cannot be detected against the existing variability of those radionuclides in the environment.

Co-60 is the only radionuclide reported in all the measurements performed in the environment around all the studied NPPs which—due to its short radioactive half-life—is expected to be present in the environment exclusively due to the contribution of discharges from nuclear facilities, in the absence of other sources discharging this radioisotope to the environment. Therefore, this is the only radionuclide which could be used as indicator in this study.

Given the conservatism of the models used for the study, the results of the prospective assessments were expected to be above concentrations measured in the environment (around one order of magnitude). The modelled values for Co-60 showed a reasonable correspondence with measured environmental data in all cases, being however conservative only in two of them. The more conservative predictions obtained with the models were the activity concentrations in the sediments of a lake (Almaraz) where, on average, values two times higher were obtained (51 Bq/kg modelled against 26 Bq kg^−1^ measured). For the river (Ascó), calculated results were adequately conservative—up to 3.4 times on average (4.2 Bq kg^−1^ modelled against 1.25 Bq kg^−1^ measured). In this case of a river, the importance of location should be pointed out, as when not conservatively selected, overestimations cannot always be assured. Finally, for coasts (Vandellos II), prospective results were in the same range as the environmental measurements, obtaining predictions that were at maximum 1.1 times higher than measured values. Therefore, for the case of the coastal model it can be established that the models were not conservative enough, although the results, on average, were relatively close to the real values.

The scarcity of measured data and information on the location of sampling points did not allow a more precise comparison of prospective assessments with measured values. However, this was a good exercise for testing the degree of conservatism of generic models applied in specific conditions. Carrying out similar exercises using a greater number of measured values, lower detection limits, and precise information on the sampling would be beneficial for further comparisons.

## Figures and Tables

**Table 1:** Data of triangular distributions used for the nuclear power plants’ (NPPs’) liquid releases and for the main radionuclides, provided for the period 1999–2003 [5].

**Almaraz NPP (Dammed Reservoir)**			
**Release Type**	**Radionuclide**	**Parameter**	**Value (Bq s^−1^)**
Liquid	Co-60	Minimum	39
		Mean	54
		Maximum	66
	Cs-137	Minimum	15
		Mean	26
		Maximum	35
**Vandellós II NPP (Coastal Waters)**			
**Release Type**	**Radionuclide**	**Parameter**	**Value (Bq s^−1^)**
Liquid	Co-60	Minimum	39
		Mean	54
		Maximum	66
	Cs-137	Minimum	15
		Mean	26
		Maximum	35
	Sr-90	Minimum	43
		Mean	73
		Maximum	160
**Ascó NPP (River)**			
**Release Type**	**Radionuclide**	**Parameter**	**Value (Bq s^−1^)**
Liquid	Co-60	Minimum	34
		Mean	51
		Maximum	72
	Cs-137	Minimum	5
		Mean	23
		Maximum	36
	Sr-90	Minimum	8
		Mean	21
		Maximum	51

**Table 2:** Modelled results of radionuclide concentrations in bottom sediments (Cbs) due to Almaraz NPP’s liquid releases and range of reported measurement values (CSN, 2006) [12].

	Model			Measurement
Radionuclide	C_bs_ (Bq kg^−1^) Mean	C_bs_ (Bq kg^−1^) Standard Deviation	C_bs_ (Bq kg^−1^) 95% Confidence Interval	Range of Measured Results (Bq kg^−1^)
Co-60	51	38	(3.6–131)	(12–40)
Cs-137	127	62	(87–130)	(11–112)

**Table 3:** Modelled results obtained for radionuclide concentrations in bottom sediments (C_bs_) due to liquid releases from Vandellós II NPP and the range of reported measurement values [12].

Hypothetical Calculation Point	Radionuclide	C_bs_ (Bq kg^−1^) Mean	C_bs_ (Bq kg^−1^) 95% Confidence Interval	Range of Measured Results (Bq kg^−1^)
Sea 1 x = 500 m y = 50 m	Co-60	0.8	(0.5–1.0)	(0.3–1.1)
	Cs-137	2.1 × 10^−2^	(1.2 × 10^−2^–3.3 × 10^−2^)	(0.7–10)
	Sr-90	6.1 × 10^−4^	(1.9 × 10^−4^–1.1 × 10^−3^)	(1–3)
Sea 2 x = 1000 m y = 100 m	Co-60	0.8	(0.5–1.1)	(0.3–1.1)
	Cs-137	2.2 × 10^−2^	(1.3 × 10^−2^–3.4 × 10^−2^)	(0.7–10)
	Sr-90	6.3 × 10^-4^	(2.0 × 10^−4^–1.1 × 10^−3^)	(1–3)
Sea 3 x = 2000 m y = 200 m	Co-60	0.7	(0.4–0.9)	(0.3–1.1)
	Cs-137	1.9 × 10^-2^	(1.1 × 10^−2^–2.9 × 10^−2^)	(0.7–10)
	Sr-90	5.4 × 10^−4^	(1.7 × 10^−4^–9.7 × 10^−4^)	(1–3)
Sea 4 x = 5000 m y = 500 m	Co-60	0.5	(0.3–0.6)	(0.3–1.1)
	Cs-137	1.3 × 10^−2^	(7.4 × 10^−3^–1.9 × 10^−2^)	(0.7–10)
	Sr-90	3.6 × 10^−4^	(1.2 × 10^−4^–6.5 × 10^−4^)	(1–3)

**Table 4:** Modelled results obtained for radionuclide concentrations in bottom sediments (C_bs_) due to liquid releases from Ascó NPP and the range of reported measured values [12]. Note: S—same shore; D—different shore.

Hypothetical Calculation Point	Radionuclide	C_bs_ (Bq kg^−1^) Mean	C_bs_ (Bq kg^−1^) 95% Confidence Interval	Range of Measured Results (Bq kg^−1^)
River 1 d = 500 m S	Co-60	4.2	(0.49–7.8)	(0.5–2)
	Cs-137	1.09	(6.82 × 10^−2^–3.13)	(1–8)
	Sr-90	6.2 × 10^−2^	(2.9 × 10^−3^–2.9 × 10^−1^)	(0.8–2)
River 2 d = 500 m D	Co-60	0.8	(9.4 × 10^−2^–1.5)	(0.5–2)
	Cs-137	0.21	(1.3 × 10^−2^–0.6)	(1–8)
	Sr-90	1.2 × 10^−2^	(5.7 × 10^−4^–5.7 × 10^−2^)	(0.8–2)
River 3 d = 1000 m S	Co-60	3.3	(0.4–6)	(0.5–2)
	Cs-137	0.9	(5.3 × 10^−2^–2.5)	(1–8)
	Sr-90	4.8 × 10^−2^	(2.3 × 10^−3^–0.23)	(0.8–2)
River 4 d = 2000 m S	Co-60	2.5	(0.3–4.7)	(0.5–2)
	Cs-137	0.6	(4.1 × 10^−2^–1.9)	(1–8)
	Sr-90	3.7 × 10^−2^	(1.8 × 10^−3^–0.2)	(0.8–2)
River 5 d = 2000 m D	Co-60	0.8	(9.4 × 10^−2^–1.5)	(0.5–2)
	Cs-137	0.2	(1.3 × 10^−2^–0.6)	(1–8)
	Sr-90	1.2 × 10^−2^	(5.7 × 10^−4^–5.6 × 10^−2^)	(0.8–2)
